# Supplementary figures and images for: Interactions of SARS-CoV-2 envelope protein with amilorides correlate with antiviral activity
Source: PLoS Pathog. 2021 May 18;17(5):e1009519. doi: 10.1371/journal.ppat.1009519 (PMC8184013; doi:10.1371/journal.ppat.1009519)

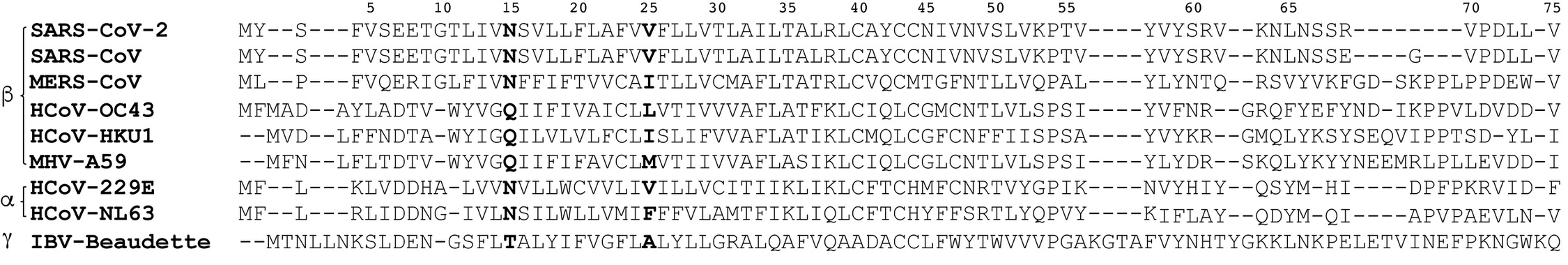

Supplement: S1 Fig — Three subgroups are indicated. The numbers at the top of the amino acid sequence corresponds to SARS-CoV-2 E protein. (TIF) [file ppat.1009519.s001.tif]

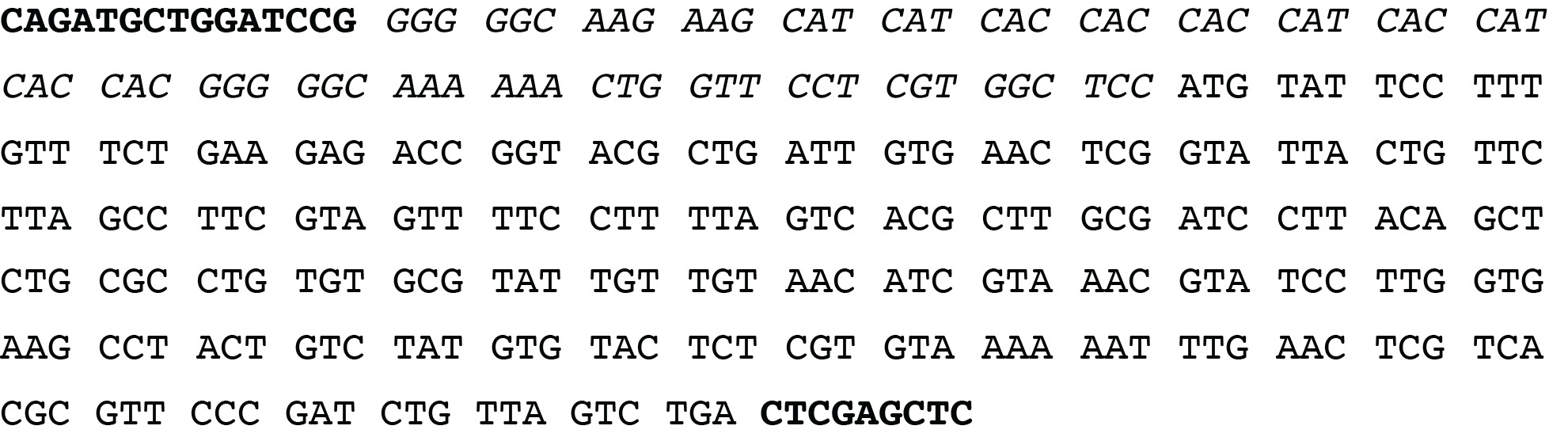

Supplement: S2 Fig — The N-terminal linker sequence containing a ten histidine tag and a thrombin cleavage site is shown in italics. The sequences in bold contain the multiple restriction sites for cloning. AlwNI and XhoI sites were inserted for KSI-fusion system with pET31b(+) vector (www.emdmillipore.com). BamHI and SacI sites were inserted for GST-fusion system using pGEX-2T vector (www.sigmaaldrich.com). (TIF) [file ppat.1009519.s002.tif]

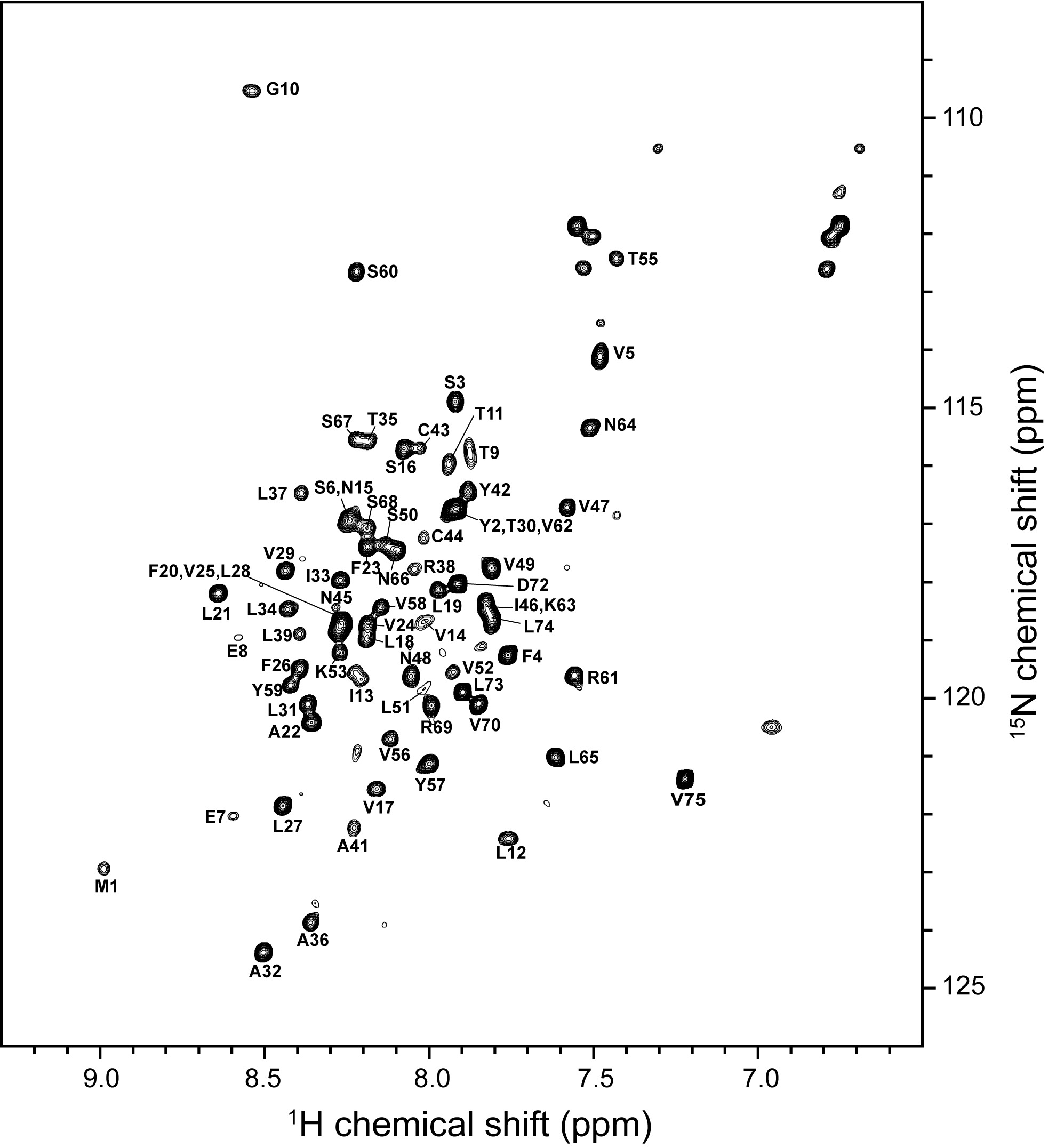

Supplement: S3 Fig — The spectrum was obtained at a 1H resonance frequency of 800 MHz Resonance assignments are marked. (TIF) [file ppat.1009519.s003.tif]

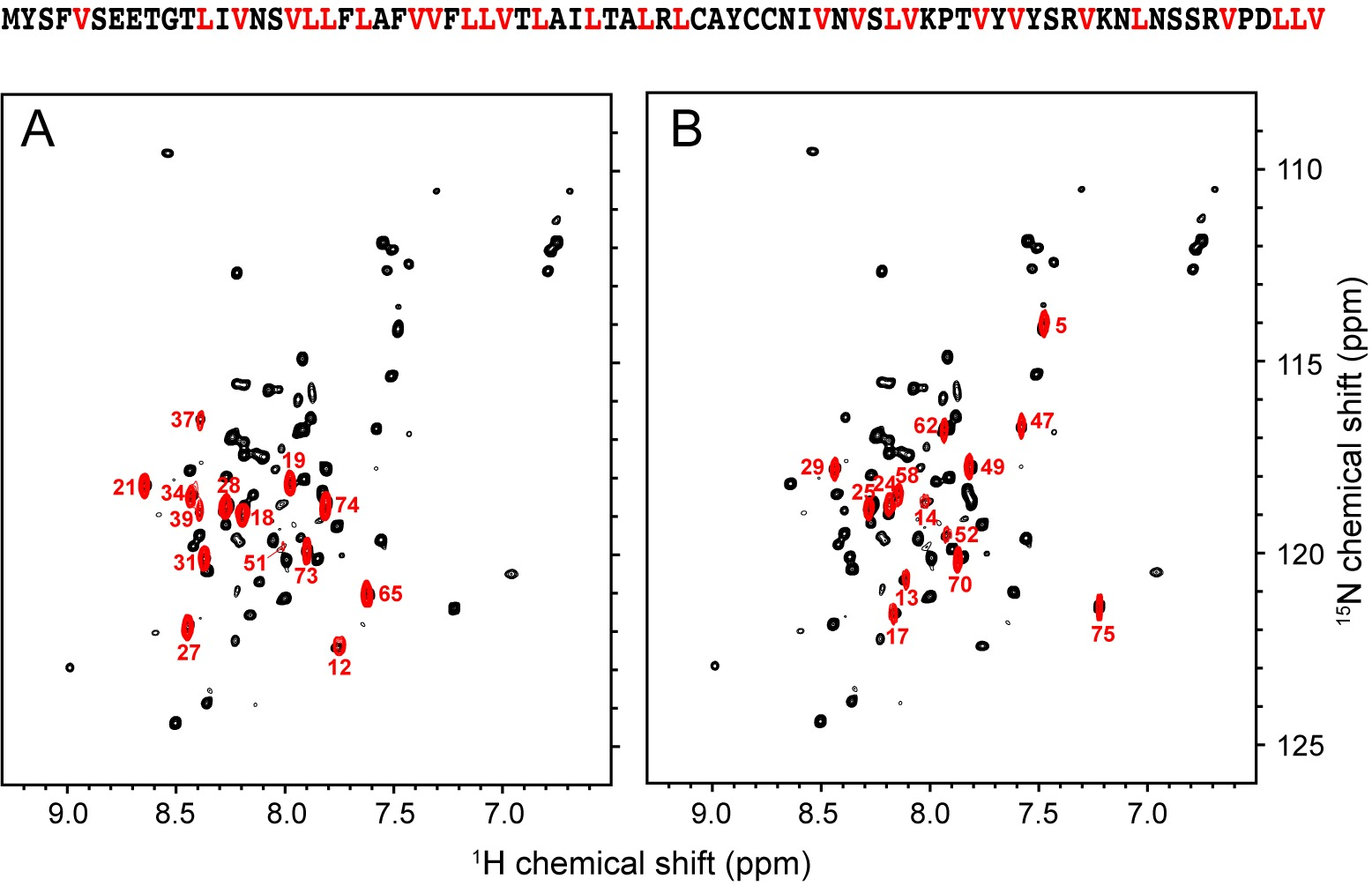

Supplement: S4 Fig — A. 15N-Leu labeled EF. B. 15N-Val labeled EF. The spectra of selectively labeled EF (red contours) are superimposed on that of uniformly labeled EF (black contours). Resonance assignments of the selectively labeled spectra are marked. The positions of the leucine and valine residues are indicated in red in the sequence of E protein. (TIF) [file ppat.1009519.s004.tif]

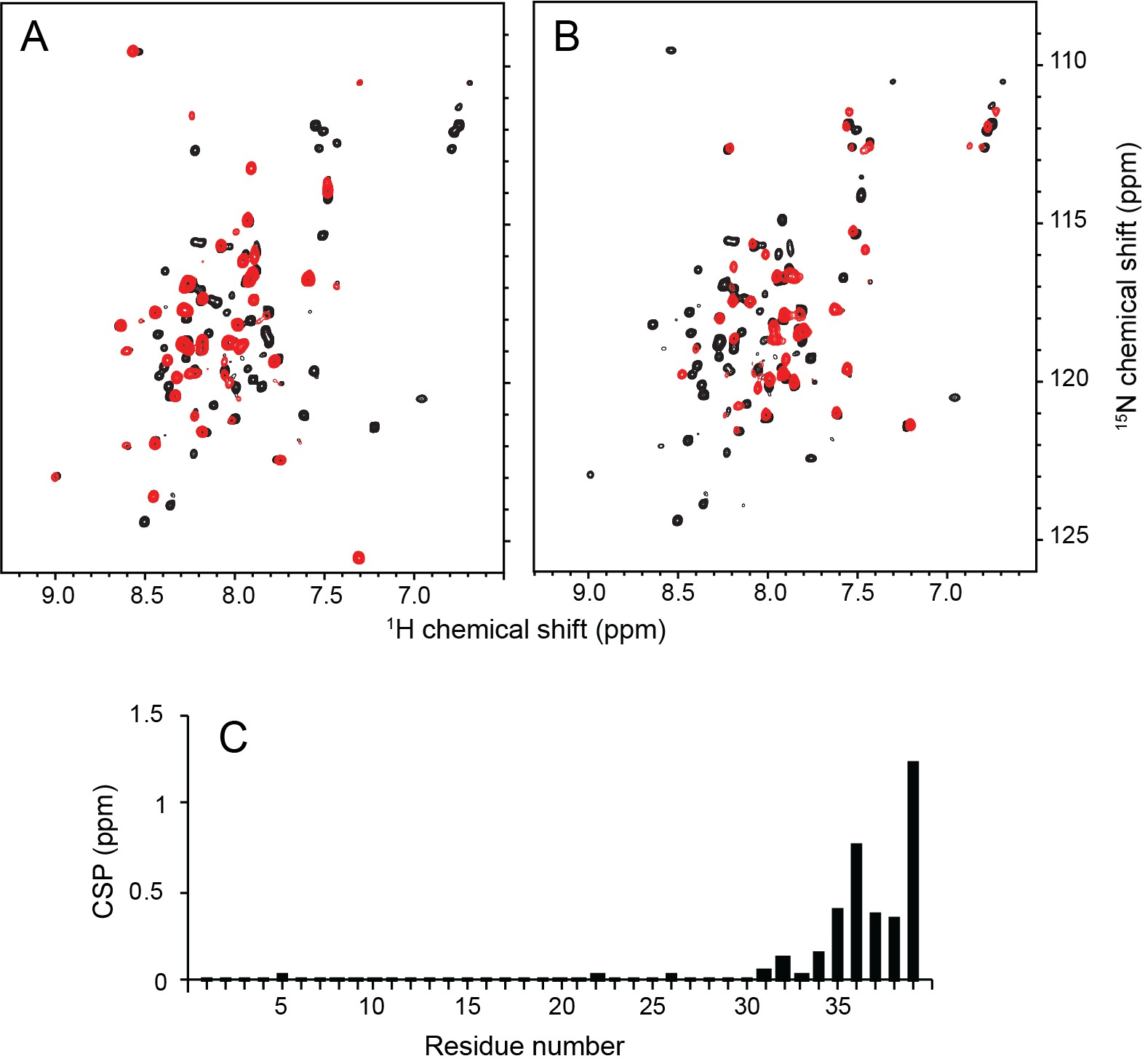

Supplement: S5 Fig — A. E protein transmembrane domain (ET) (residues 1–39). B. E protein cytoplasmic domain (EC) (residues 36–75). The spectra of the truncated constructs (red contours) are superimposed on that of the full-length E protein (EF) (black contours). C. Chemical shift perturbation plot of ET resonance frequencies compared to those of EF as a function of residue number. (TIF) [file ppat.1009519.s005.tif]

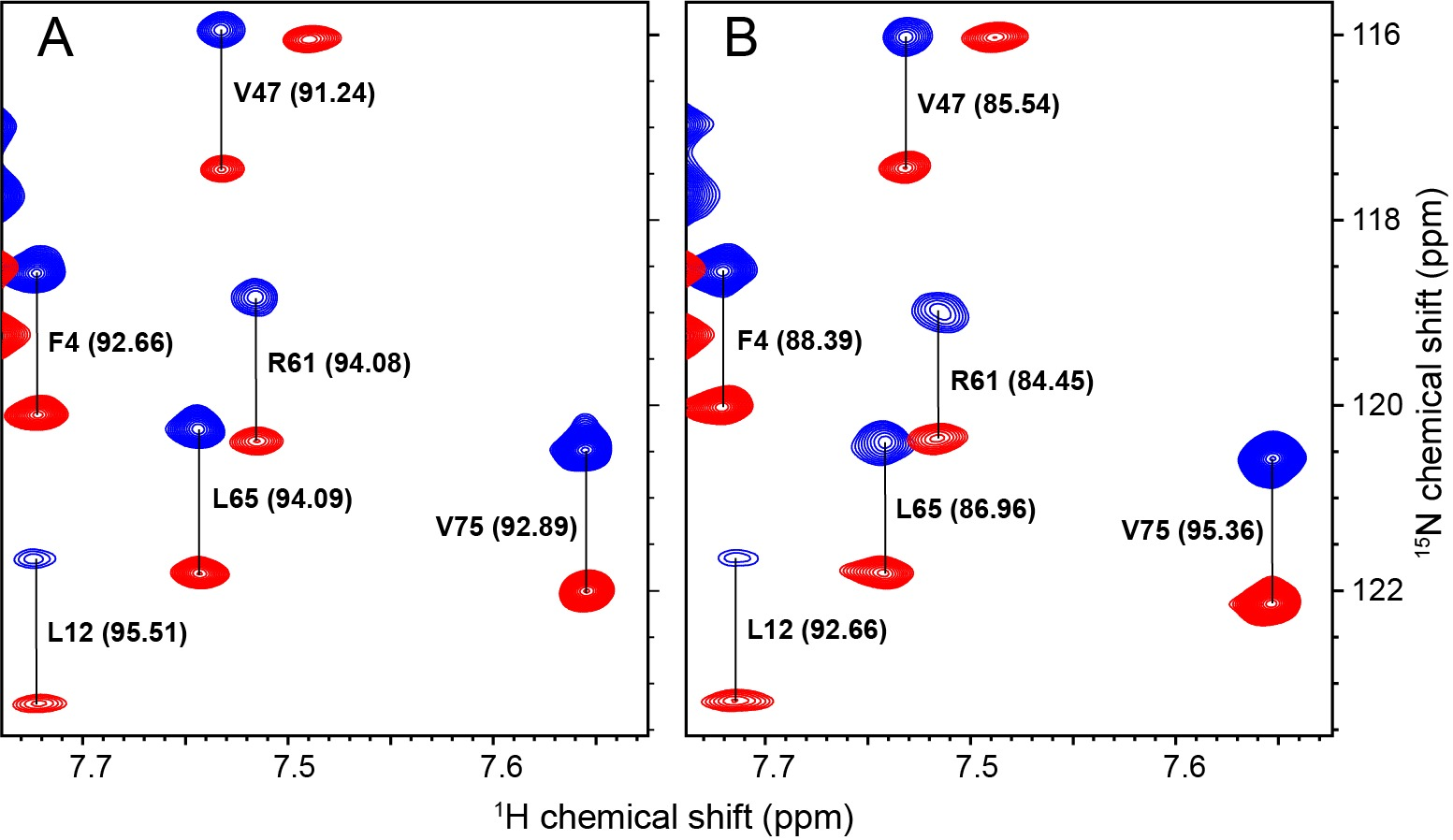

Supplement: S6 Fig — A. Isotropic sample. B. Weakly aligned sample in the presence of Y21M fd bacteriophage at 20 mg/mL. Residue numbers and 1JNH couplings are indicated in parenthesis, respectively. (TIF) [file ppat.1009519.s006.tif]

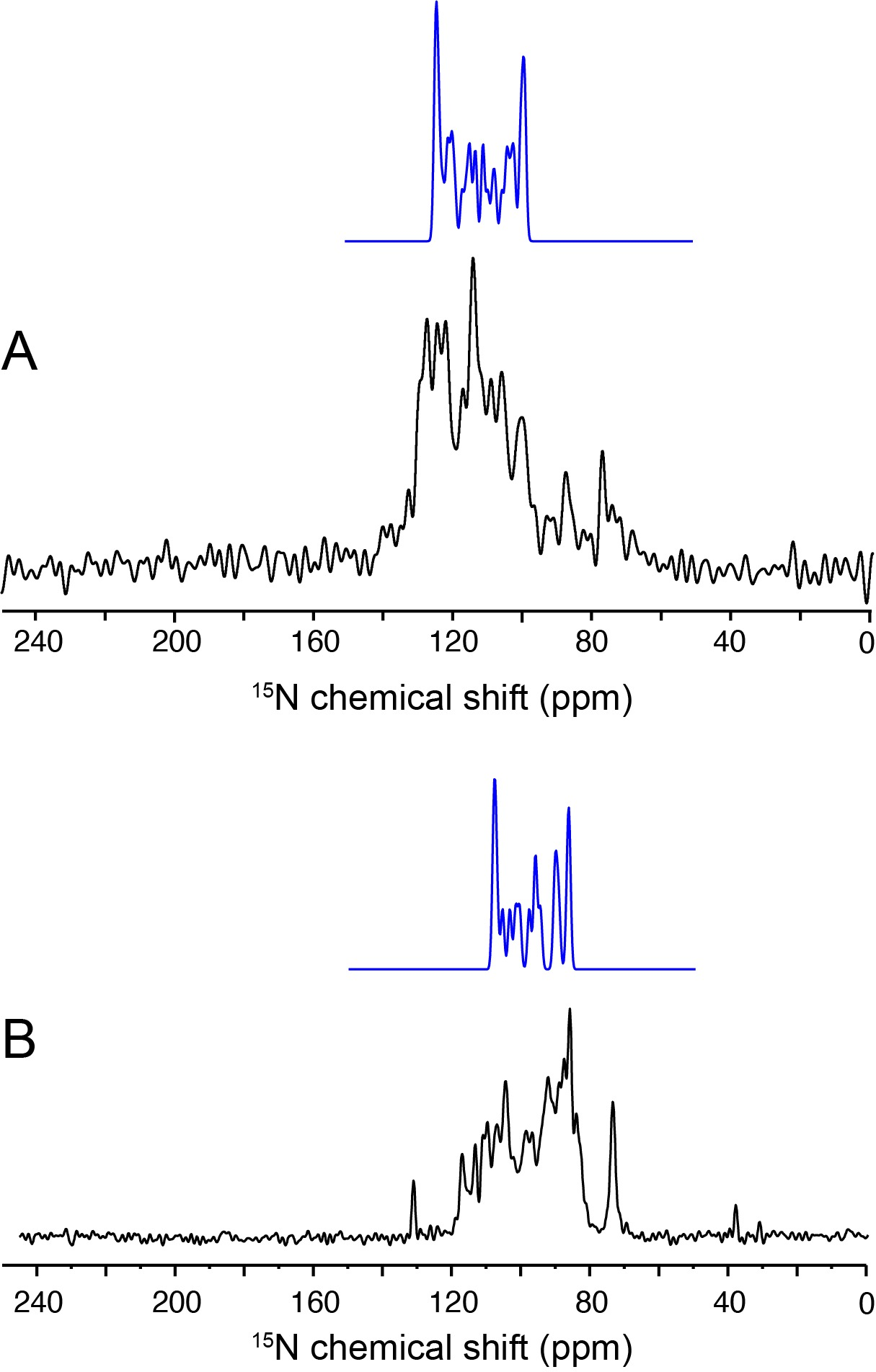

Supplement: S7 Fig — A. Transmembrane domain of E protein (ET) from SARS-CoV-2. B. Transmembrane domain of Virus Protein U (VPU) from HIV-1 [77]. The spectrum of ET sample was obtained at 35°C on a Bruker 900 MHz spectrometer using a home-built 1H/15N double-resonance probe with a MAGC coli for the 1H channel and a solenoid coil for the 15N channel [78]. Uniformly 15N-labeled ET was embedded in 1,2-dimyristoyl-sn-glycero-phosphocholine (DMPC) bilayers oriented with the lipid bilayer normal perpendicular to the applied magnetic field. The molar ratio of DMPC to ET is 395:1 and the DMPC concentration is 30% (w/v). Fast uniaxial rotational diffusion of both proteins about the bilayer normal yielded motionally averaged single line resonances. The spectra in blue are 15N chemical shift projections of the two-dimensional calculated PISA wheel spectra [58,59] with (A) a 36-residue ideal helix (PHI = -61o and PSI = -45o) with its helix axis tilted 45o from the lipid bilayer normal and (B) a 18-residue ideal helix (PHI = -61o and PSI = -45o) with its helix axis tilted 30o from the lipid bilayer normal. (TIF) [file ppat.1009519.s007.tif]
