# Supplementary material for: Interactions of SARS-CoV-2 envelope protein with amilorides correlate with antiviral activity
Source: PLoS Pathog. 2021 May 18;17(5):e1009519. doi: 10.1371/journal.ppat.1009519 (PMC8184013; doi:10.1371/journal.ppat.1009519)
Supplement: S1 Table — (DOCX) [file ppat.1009519.s008.docx]

| Resid | number | NH | 15N | CA shift | CO shift | NH RDC |
| --- | --- | --- | --- | --- | --- | --- |
| M | 1 | 8.990 | 122.931 | 57.773 | 176.089 | -2.86 |
| Y | 2 | 7.919 | 116.747 | 58.893 | 176.390 |  |
| S | 3 | 7.921 | 114.872 | 60.298 | 174.761 | 1.43 |
| F | 4 | 7.760 | 119.246 | 59.365 | 175.286 | -4.28 |
| V | 5 | 7.481 | 114.106 | 62.140 | 175.198 | 1.42 |
| S | 6 | 8.242 | 116.926 | 58.808 | 175.103 |  |
| E | 7 | 8.598 | 122.019 | 58.719 | 178.061 | -1.42 |
| E | 8 | 8.577 | 118.929 | 58.580 | 176.853 | -1.34 |
| T | 9 | 7.876 | 115.829 | 65.811 | 175.949 | 6.25 |
| G | 10 | 8.540 | 109.520 | 47.297 | 174.952 | 7.66 |
| T | 11 | 7.939 | 115.917 | 65.742 | 175.523 | 0.01 |
| L | 12 | 7.762 | 122.407 | 58.262 | 179.461 | -2.85 |
| I | 13 | 8.225 | 119.551 | 65.514 | 176.963 | 5.47 |
| V | 14 | 8.017 | 118.650 | 67.265 | 177.078 | 2.84 |
| N | 15 | 8.242 | 116.926 | 56.111 | 177.326 |  |
| S | 16 | 8.077 | 115.681 | 63.352 | 175.469 | 2.07 |
| V | 17 | 8.160 | 121.552 | 67.144 | 177.586 | 9.48 |
| L | 18 | 8.190 | 118.966 | 58.621 | 178.348 | 0.55 |
| L | 19 | 7.970 | 118.093 | 58.285 | 177.894 | -1.45 |
| F | 20 | 8.268 | 118.695 | 61.855 | 176.951 |  |
| L | 21 | 8.643 | 118.184 | 58.035 | 178.178 | 5.70 |
| A | 22 | 8.360 | 120.410 | 55.612 | 178.456 | 1.43 |
| F | 23 | 8.187 | 117.385 | 61.528 | 176.581 | 2.31 |
| V | 24 | 8.189 | 118.727 | 67.483 | 177.894 | 4.86 |
| V | 25 | 8.268 | 118.695 | 67.829 | 177.045 |  |
| F | 26 | 8.395 | 119.490 | 61.733 | 178.105 | -2.85 |
| L | 27 | 8.446 | 121.848 | 58.304 | 177.894 | 1.77 |
| L | 28 | 8.268 | 118.695 | 58.415 | 178.617 |  |
| V | 29 | 8.438 | 117.791 | 67.201 | 178.436 | 2.85 |
| T | 30 | 7.881 | 116.432 | 68.663 | 176.412 |  |
| L | 31 | 8.370 | 120.095 | 58.144 | 179.838 | 5.70 |
| A | 32 | 8.505 | 124.376 | 55.910 | 178.692 | 4.28 |
| I | 33 | 8.272 | 117.959 | 65.871 | 177.718 | -0.01 |
| L | 34 | 8.432 | 118.455 | 58.573 | 178.851 | 4.28 |
| T | 35 | 8.186 | 115.567 | 68.218 | 176.246 | 9.97 |
| A | 36 | 8.361 | 123.858 | 55.799 | 179.377 | 5.70 |
| L | 37 | 8.389 | 116.444 | 57.949 | 178.649 | 1.42 |

| Resid | number | NH | 15N | CA shift | CO shift | NH RDC |
| --- | --- | --- | --- | --- | --- | --- |
| R | 38 | 8.047 | 117.764 | 57.985 | 178.919 | 8.52 |
| L | 39 | 8.394 | 118.863 | 57.077 |  | 8.56 |
| C | 40 |  |  | 61.977 | 175.327 |  |
| A | 41 | 8.230 | 122.237 | 54.581 | 178.267 | -1.82 |
| Y | 42 | 7.884 | 116.422 | 59.824 | 176.854 | 7.33 |
| C | 43 | 8.031 | 115.681 | 60.727 | 174.876 | 2.99 |
| C | 44 | 8.019 | 117.248 | 60.158 |  | -5.29 |
| N | 45 | 8.300 | 118.418 | 54.323 | 175.567 |  |
| I | 46 | 7.828 | 118.354 | 62.515 | 175.562 |  |
| V | 47 | 7.581 | 116.702 | 62.700 | 175.174 | -5.70 |
| N | 48 | 8.056 | 119.603 | 53.545 | 175.154 | -8.56 |
| V | 49 | 7.810 | 117.742 | 62.629 | 175.254 | 1.42 |
| S | 50 | 8.135 | 117.375 | 58.845 |  | -0.06 |
| L | 51 | 8.012 | 119.860 | 55.800 | 177.905 |  |
| V | 52 | 7.927 | 119.544 | 66.117 | 176.475 |  |
| K | 53 | 8.272 | 119.204 | 60.393 |  | -6.76 |
| P | 54 |  |  | 65.890 | 177.282 |  |
| T | 55 | 7.432 | 112.404 | 67.457 | 176.185 | -8.55 |
| V | 56 | 8.117 | 120.700 | 66.620 | 178.318 | -11.40 |
| Y | 57 | 8.000 | 121.121 | 61.674 | 178.017 | -4.28 |
| V | 58 | 8.146 | 118.426 | 67.393 | 177.426 | -1.36 |
| Y | 59 | 8.422 | 119.762 | 61.661 | 176.551 | -12.83 |
| S | 60 | 8.220 | 112.631 | 61.706 | 175.756 | -8.55 |
| R | 61 | 7.558 | 119.592 | 57.242 | 177.417 | -9.63 |
| V | 62 | 8.019 | 117.248 | 64.973 | 177.119 |  |
| K | 63 | 7.828 | 118.354 | 58.385 | 176.704 |  |
| N | 64 | 7.515 | 115.330 | 53.511 | 175.608 | -9.99 |
| L | 65 | 7.616 | 121.004 | 56.176 | 176.465 | -7.13 |
| N | 66 | 8.096 | 117.428 | 53.369 | 175.375 | -5.35 |
| S | 67 | 8.224 | 115.539 | 59.418 | 174.884 | 0.00 |
| S | 68 | 8.189 | 117.062 | 59.493 | 174.269 | 5.78 |
| R | 69 | 7.994 | 120.114 | 55.850 | 175.984 | -5.70 |
| V | 70 | 7.850 | 120.081 | 62.872 |  | 0.01 |
| P | 71 |  |  | 64.130 | 176.190 |  |
| D | 72 | 7.914 | 118.016 | 55.115 | 176.190 | 2.85 |
| L | 73 | 7.898 | 119.889 | 55.726 | 176.420 | -2.85 |
| L | 74 | 7.809 | 118.626 | 55.168 | 175.580 |  |
| V | 75 | 7.222 | 121.375 | 63.256 |  | 2.47 |
