# Supplementary material for: Interactions of SARS-CoV-2 envelope protein with amilorides correlate with antiviral activity
Source: PLoS Pathog. 2021 May 18;17(5):e1009519. doi: 10.1371/journal.ppat.1009519 (PMC8184013; doi:10.1371/journal.ppat.1009519)
Supplement: Attachment — Submitted filename: Opella Plos Pathogens Review.docx [file ppat.1009519.s009.docx]

Review -Opella et al., PlosPathogens manuscript review

The full-length protein is solubilized in detergent micelles for solution NMR structural characterization. This approach could be fraught with potential structural distortions from the weak modeling of the membrane environment. Solution NMR spectroscopy in nanodiscs would be much better, but more time consuming and urgency is needed. Overall this manuscript represents an unusual mix of expertise, skills, experiments and conclusions that needs to be available to the scientific community as soon as possible.

One of the primary questions from earlier work would have been to confirm the oligomerization state. While an earlier study claimed that it is a pentamer there was little evidence supporting that claim and even less evidence for claiming that the hydrophobic pore was an ion channel. Here the authors acknowledge that this is unreasonable, but do not resolve the oligomerization state, nor address the structural role of the three cysteine residues at the aqueous C-terminus of the TM helix. It seems to me that this characterization of the structure is important prior to gaining any understanding of how this protein functions. The authors claim that careful sample preparation is the key – I agree, and I do not doubt that the authors have a structured state that is uniform across their sample – but it is not clear what that state is.

The authors have done a superb job of expressing in significant quantities the E protein. The protein runs as a monomer in HPC micelles on SDS Page gels and all of the studies performed here appear to be conducted with this monomeric preparation. To think that this protein is a monomer in the virion is possible as the TM helix has only a single weakly hydrophilic residue, a threonine, in the middle of the TM helix. However, if the authors think the TM domain is much longer, such as 35 residues then do they think it is still a monomer. Of course the detergent micelle might favor a monomeric version of the protein. The N-H exchange experiments identify a hydrophobic core of residues of 14-16 residues rather typical of helices and not suggestive of an extra-long TM helix. Do I gather from the supplemental information that the authors think the TM helix would be tilted at 45° to the bilayer normal and hence a lot more embedded in a hydrocarbon environment than is likely in the micelle environment?

The RDC alignment was induced by filamentous viral particles and displays a good oscillatory behavior for a long helix. Units for the coupling should be shown. The ratios of intensity with and without D2O suggest that the TM domain of the helix is not excessively long and hence the tilt angle with the micellar axis and the presumed bilayer normal would not be large.

HMA binding is clearly to the N-terminus involving residues very close to the N-terminus up through residue 18, the start of the TM. One of the problems with using micelles is the N and C termini are not necessarily well separated, especially in a situation where there is not a lot of structure in the C-terminus. Some of the compounds with bulky substituents were shown to have sub µM IC-50’s. Both HMA and EIPA were found to be effective and apparently act late in the viral life cycle.

Co-expression of viral proteins M and N in HEK cells resulted in some viral-like particles. The number of these particles increased once E protein was also expressed.

A couple of mutations for the E protein were studied N15A and V25F – not surprisingly N15A in the N-terminus resulted in changes and V25F resulted in little change. Apparently, it had been previously demonstrated that ion channel activity by E protein had been influenced by these mutations. Since there is no way that this protein with its extremely hydrophobic TM helix is an ion channel these current results are not surprising. Apparently N15 is essential for drug binding as N15A greatly reduced drug binding.

It is only in the discussion that the authors mention the three Cys residues. What was done to prevent disulfide linkages between proteins?

The study of covid proteins is a special and urgent task – under normal circumstances the mix of so many different experiments leading to a wide range of conclusions could be criticized as unfocused, rambling, etc., but this work on CoE has special and immediate importance to the scientific community. I would therefore recommend publishing – despite the choice of detergent micelles, which is clearly non-optimal there is a lot of good results here that should be visible to the broad scientific community. Maybe the most important point is the lack of an evidence that this protein forms an ion channel or that it forms an oligomeric state.
