# Supplementary material for: Interactions of SARS-CoV-2 envelope protein with amilorides correlate with antiviral activity
Source: PLoS Pathog. 2021 May 18;17(5):e1009519. doi: 10.1371/journal.ppat.1009519 (PMC8184013; doi:10.1371/journal.ppat.1009519)
Supplement: Attachment — Submitted filename: Response to Reviewers.docx [file ppat.1009519.s010.docx]

**Response to Reviewers Comments (PPATHOGENS-D-21-00658)**

**Reviewer 1**

**Comment 1:** It is only in the discussion that the authors mention the three Cys residues. What was done to prevent disulfide linkages between proteins?

**Response 1**: In the Materials and Methods section, we described that the reducing agent Tris (2-carboxyethyl) phosphine hydrochloride (TCEP-HCl) was present at a concentration of 10 mM in the samples of both the full-length E protein (EF) and the C-terminal domain (EC) in order to ensure that the cysteine residues remain reduced. We did not observe any significant changes in the NMR spectra during the course of the experiments. Formation of intra- or inter- molecular disulfide bonds would have surely altered the spectra.

**Reviewer 2**

**Comment 1:** Describe method to transfer protein into 90% D2O.

**Response 1**: We have included the exchange procedure of the NMR samples in 90% H_2_O with 90% D_2_O in the Materials and Methods: “For H/D exchange experiments, the 90% D_2_O NMR samples were prepared by 9-fold dilution of the samples in 90% H_2_O/10% D_2_O with a 100% D_2_O NMR buffer and filter concentration with a 10 kDa molecular weight cut-off filter.”

**Comment 2:** Use consistent nomenclature: D_2_O in text – ^2^H_2_O in methods.

**Response 2**: We have changed ^2^H_2_O to D_2_O where necessry for consistency throughout the manuscript.

**Comment 3:** The HPC concentration, if known accurately, may be low for this protein. The protein concentration = 500 mM (100 mM pentamer). HPC is estimated to be 123 mM or 172 mM after concentration by filtration (is this assuming that detergent does not pass through the filter? – if so, then this is an upper limit). Even without knowledge of the aggregation number of HPC containing E-protein, the ratio of pentamer to detergent monomer is 1: 1.23 – 1.72. Ideally, there should be at least one micelle per pentamer – but this ratio is unlikely under these conditions. It is possible that E-protein has adsorbed more HPC during the purification/proteolysis method. Can the authors report how the HPC concentration was determined? A direct measurement would be most useful – however, if this is not possible, simply adding detergent to an EF sample and observing if NMR signals change would be useful to demonstrate that the structural features found are not a consequence of “artifactual togetherness” owing to a limited number of micelles.

**Response 3**: We have included the measurement of HPC concentration in E protein samples in the Materials and Methods section: “The HPC concentration in the E protein NMR samples was estimated by comparison of the ^1^H NMR signal intensity from the HPC acyl chains with that from a 5% (w/v) (123 mM) HPC reference sample. HPC with its low critical micelle concentration and high aggregation number did not pass through the membrane filter with a 10 kDa molecular weight cut-off filter. Although the HPC concentration varied slightly batch to batch after filter concentration, no significant changes of NMR spectra were observed for HPC:E protein monomer molar ratio between about 100:1 and 250:1. Nevertheless, in order to maintain a consistent ratio of HPC to E protein in the sample, the HPC concentration was adjusted to 5% (w/v) for 0.5 mM E protein and 7% (w/v) for 1 mM E protein samples, respectively.”

**Revisions**

The minor revisions that address the concerns of the reviewers are highlighted in yellow. A clean version of the manuscript is also submitted.
